# Supplementary material for: Development of the Digital Assessment of Precise Physical Activity (DAPPA) Tool for Older Adults
Source: Int J Environ Res Public Health. 2020 Oct 29;17(21):7949. doi: 10.3390/ijerph17217949 (PMC7663633; doi:10.3390/ijerph17217949)
Supplement: Supplementary file 1 [file ijerph-17-07949-s001.pdf]

Table S1. Activities available to select in DAPPA Tool.

Activity	METs	Ainsworth (2011) Compendium of Physical Activity Code
**Home and Garden**		
Light Gardening (e.g., light weeding, trimming shrubs)	3.5	08239
Heavy Gardening (e.g., heavy weeding, shovelling)	5.0	08241
Ironing	1.8	05070
Lawn mowing	4.5	08125
Cooking	2	05050
Light Housework (e.g., sweeping, vacuuming)	2.8	05025
Heavy Housework (e.g., washing windows, cleaning gutters)	3.3	05030
Laundry	2	05090
Caring for a disabled partner/family member	4	05200
Working on car	3.3	06030
Home repair (e.g., DIY, decorating)	4.5	06127
Walking the dog	3	17165
**Out and About**		
Leisurely Walking (e.g., around town, to the doctors)	3.5	17160
Brisk Walking (e.g., to the shops, around town)	4.3	17200
Leisurely Cycling	3.5	01018
Cycling	7.5	01015
Walking the dog	3	17165
Shopping	3.3	05060
Carrying shopping	2.5	05055
Volunteering	3	21070
Driving	2.5	16010
Travelling on public transport (e.g., train or bus)	1.3	16016
Labouring	4.0	11120
Custodial work	2.3	11125
Office work	1.5	11580
Retail work	3	11600
**Sports and Exercise**		
Leisurely Walking	3.5	17160
Brisk Walking	4.3	17200
Hiking	6	17080
Cycling (Leisurely)	3.5	01018
Cycling	7.5	01015
Archery	4.3	15010
Badminton	5.5	15030
Basketball	6.5	15055
Snooker/Pool	2.5	15080
Bowling	3	15090
Canoeing/Kayaking	3.5	18070
Cricket	4.8	15150
Croquet	3.3	15160
Exercise Bike (Stationary)	7.0	02010
Exercise class	5	02061
Football	8	15230
Golf (Walking)	4.3	15255
Golf (Using a cart)	3.5	15290
Hockey	7.8	15350

Horse riding	5.5	15370
Jogging	7	12020
Light calisthenics (e.g., situps, abdominal crunches)	2.8	02024
Moderate calisthenics (e.g., push-ups, sit ups, pull-ups)	3.8	02022
Martial Arts (gentle practice)	5.3	15425
Martial Arts (vigorous practice/sparring)	10.3	15430
Mountain Biking	14.0	01003
Pilates	3.0	02105
Resistance (weight) training	3.5	02054
Rowing Machine	7.0	02072
Running	8	12150
Rugby	6.3	15562
Sailing	3.0	18120
Skating (Ice/Roller)	7.0	15590
Squash	12.0	15650
Swimming	5.3	18265
Table tennis	6.0	15500
Tai Chi	3	15670
Tennis (Singles)	7.3	15675
Tennis (Doubles)	4.5	15686
Water aerobics	5.5	18355
Yoga	2.5	02150
Zumba	6.5	TBD *
**Social and Leisure**		
Active video game (e.g., Wii Fit)	3.8	02003
Arts and Crafts	1.8	09075
Bowls	3.3	15465
Attending church	1.3	20000
Board Games	1.5	09000
Dancing	7.8	03031
Darts	2.5	15180
Fishing	3.5	04001
Meeting Friends	1.5	21000
Playing an Instrument	2	10074
Playing Cards	1.5	09010
Seeing Children/Grandchildren	3	09101
Sewing (machine)	2.8	05082
Sexual Activity	1.8	14020
Exercise class	5	02061

* This is as specified by the compendium—MET value is provided, but code yet to be assigned. MET, metabolic equivalent of task; DIY, do it yourself.
